# Comparative Effectiveness of Transversus Abdominis Plane (TAP) Block and Epidural Analgesia in Abdominal Surgery: A Meta-Analysis of Randomized Controlled Trials

**DOI:** 10.7759/cureus.87167

**Published:** 2025-07-02

**Authors:** Nusratun Naim, MD Selim Reza, Anwar Umar

**Affiliations:** 1 General Surgery, James Paget University Hospital, NHS Foundation Trust, Coventry, GBR; 2 General (Internal) Medicine, Oxford University Hospitals, NHS Foundation Trust, Oxford, GBR; 3 General Surgery, University Hospital Coventry and Warwickshire, Coventry, GBR

**Keywords:** abdominal surgery, pain management, tap block, thoracic epidural analgesia, vas pain score

## Abstract

Transversus abdominis plane (TAP) block and thoracic epidural analgesia (TEA) are commonly used techniques for managing postoperative pain following abdominal surgery. While TEA has traditionally been the gold standard, TAP block has emerged as a simpler, potentially safer alternative. This meta-analysis compares the efficacy and safety of TAP block versus TEA across multiple postoperative outcomes in abdominal surgery. A systematic literature search was conducted in PubMed, Embase, CENTRAL, and Scopus to identify randomized controlled trials (RCTs) that compared TAP block and TEA in adult patients undergoing abdominal surgery. Primary outcomes included pain scores at rest and during coughing at 24 and 48 hours. Secondary outcomes included postoperative opioid consumption, incidence of hypotension, time to pass first flatus, and hospital stay. Data were synthesized using a random-effects model, and heterogeneity was assessed using the *I*² statistic. Eight RCTs were included. Pain scores at rest at 24 hours showed no significant difference between TAP and TEA (mean difference [MD] = 0.26, 95% confidence interval [CI]: -0.52 to 1.03, *P* = 0.51; *I*² = 99%), as did pain scores during coughing at 24 hours (MD = 0.39, 95% CI: -0.16 to 0.94, *P* = 0.16; *I*² = 98%). At 48 hours, pain at rest remained similar (MD = 0.22, 95% CI: -0.40 to 0.85, *P* = *P* = 0.48; *I*²*I*² = 99%), while TEA showed a modest benefit during coughing (MD = 0.62, 95% CI: 0.02-1.21, *P* = 0.04; *I*² = 99%). TEA significantly reduced postoperative opioid consumption compared to TAP (MD = 8.79 mg morphine equivalent, 95% CI: 1.82-15.76, *P* = 0.01; *I*² = 82%). However, the TAP block was associated with a significantly lower risk of hypotension (risk ratio [RR] = 0.08, 95% CI: 0.01-0.40, *P* = 0.002; *I*² = 0%). No significant difference was found in time passing first flatus (MD = 4.19 hours, 95% CI: -5.22 to 13.60, *P* = 0.39; *I*² = 96%) or length of hospital stay (MD = -0.32 days, 95% CI: -1.24 to 0.60, *P* = 0.50; *I*² = 79%). TAP block provides analgesia comparable to TEA for postoperative pain at rest, with fewer hemodynamic side effects. Although TEA offers advantages in reducing opioid requirements and cough-related pain at 48 hours, its higher risk of hypotension limits its suitability in certain patients. TAP block represents an effective and safer alternative in abdominal surgical settings, particularly when TEA is contraindicated or poses a higher risk.

## Introduction and background

Managing postoperative pain effectively is crucial to enhance recovery and improve surgical outcomes. Poorly controlled pain can delay mobilization and increase the risk of postoperative complications, resulting in prolonged hospital stays. TEA has long been considered the benchmark [1] for analgesia in major abdominal operations due to its consistent efficacy in pain control and additional benefits such as reduced ileus and improved pulmonary function [1]. However, TEA is also associated with drawbacks, including hypotension, urinary retention, and the need for close monitoring [2] and technical expertise [2].

In contrast, the transversus abdominis plane (TAP) block has emerged as a simpler and less invasive alternative. By targeting the nerves supplying the anterior abdominal wall, the TAP block delivers localized somatic analgesia [3] with minimal systemic impact and preserved motor activity [3]. Its safety profile and easy use have led to widespread interest, particularly in patients where epidural analgesia is contraindicated.

Despite increasing adoption of TAP blocks, clinical studies comparing them with TEA have reported varying results. Some suggest that TAP block offers comparable analgesia with fewer side effects [4], while others report superior pain control and opioid-sparing effects with TEA [4]. Given this variability, a systematic review and meta-analysis are warranted to synthesize the existing evidence and better understand the relative benefits and limitations of these two techniques.

## Review

Methodology

Study Design

We followed Preferred Reporting Items for Systematic Reviews and Meta-Analyses (PRISMA) guidelines [5] in conducting this systematic review and meta-analysis to ensure methodological transparency [5]. The review was structured using the PICO (Population, Intervention, Comparison, Outcome) framework to ensure focused and clinically relevant questions [6].

Eligibility Criteria

The inclusion criteria encompassed adult patients (≥18 years) undergoing abdominal surgery, with studies that employed TAP block as the intervention and TEA as the comparator. Eligible trials reported at least two of the following outcomes: pain scores at rest or during coughing at 24 or 48 hours, total opioid consumption, postoperative hypotension, time to first flatus, or hospital stay. Only randomized controlled trials (RCTs) published in peer-reviewed journals were considered.

PICO criteria were incorporated as follows:

Population: Adults (≥18 years) undergoing any form of abdominal surgery.

Intervention: Use of TAP block for postoperative analgesia.

Comparison: Thoracic epidural analgesia (TEA) as the comparator technique.

Outcomes: Studies were eligible if they reported at least two of the following outcomes: postoperative pain scores at rest or during coughing at 24 and/or 48 hours; total postoperative opioid consumption; incidence of postoperative hypotension; time to first flatus (as a marker of bowel recovery); and length of hospital stay.

Study design: RCTs published in peer-reviewed journals.

Studies were excluded if they were non-randomized, observational, case reports, involved pediatric populations, focused on non-abdominal surgeries, or used other analgesic methods instead of TEA. Additionally, studies lacking extractable data or published only as abstracts or letters were excluded. 

Search Strategy

A systematic literature search was conducted in four electronic databases: PubMed, Embase, Cochrane CENTRAL, and Scopus. Search terms included a combination of keywords and MeSH terms such as “TAP block,” “transversus abdominis plane block,” “epidural analgesia,” “postoperative pain,” and “randomized controlled trial.” Manual screening of reference lists from included studies and relevant reviews was also performed to identify additional articles.

Study Selection and Data Extraction

Two independent reviewers screened all titles and abstracts, followed by full-text assessment of potentially eligible articles. Data extraction was done using a predesigned form to collect information on study characteristics, patient demographics, type of surgery, intervention details, and reported outcomes. Any disagreements were resolved through discussion or by consulting a third reviewer.

Risk-of-Bias Assessment

The risk of bias for each included RCT was assessed using the Cochrane Risk of Bias 2.0 (RoB 2) tool [7]. This tool evaluates potential bias across five domains: randomization process, deviations from intended interventions, missing outcome data, measurement of outcomes, and selection of the reported results. Each study was rated as having *low risk*, *some concerns*, or *high risk* based on domain-specific judgments.

Statistical Analysis

Data synthesis was conducted using a random-effects model due to expected clinical and methodological variability among studies. Continuous outcomes were analyzed using mean differences (MDs), and dichotomous outcomes using risk ratios (RRs), both reported with 95% CIs. Heterogeneity was quantified using the *I*² statistic, with values >50% indicating substantial heterogeneity. Where appropriate, sensitivity analyses were planned to assess the impact of individual studies on the overall results.

Results

Study Selection Process

**Figure 1:**
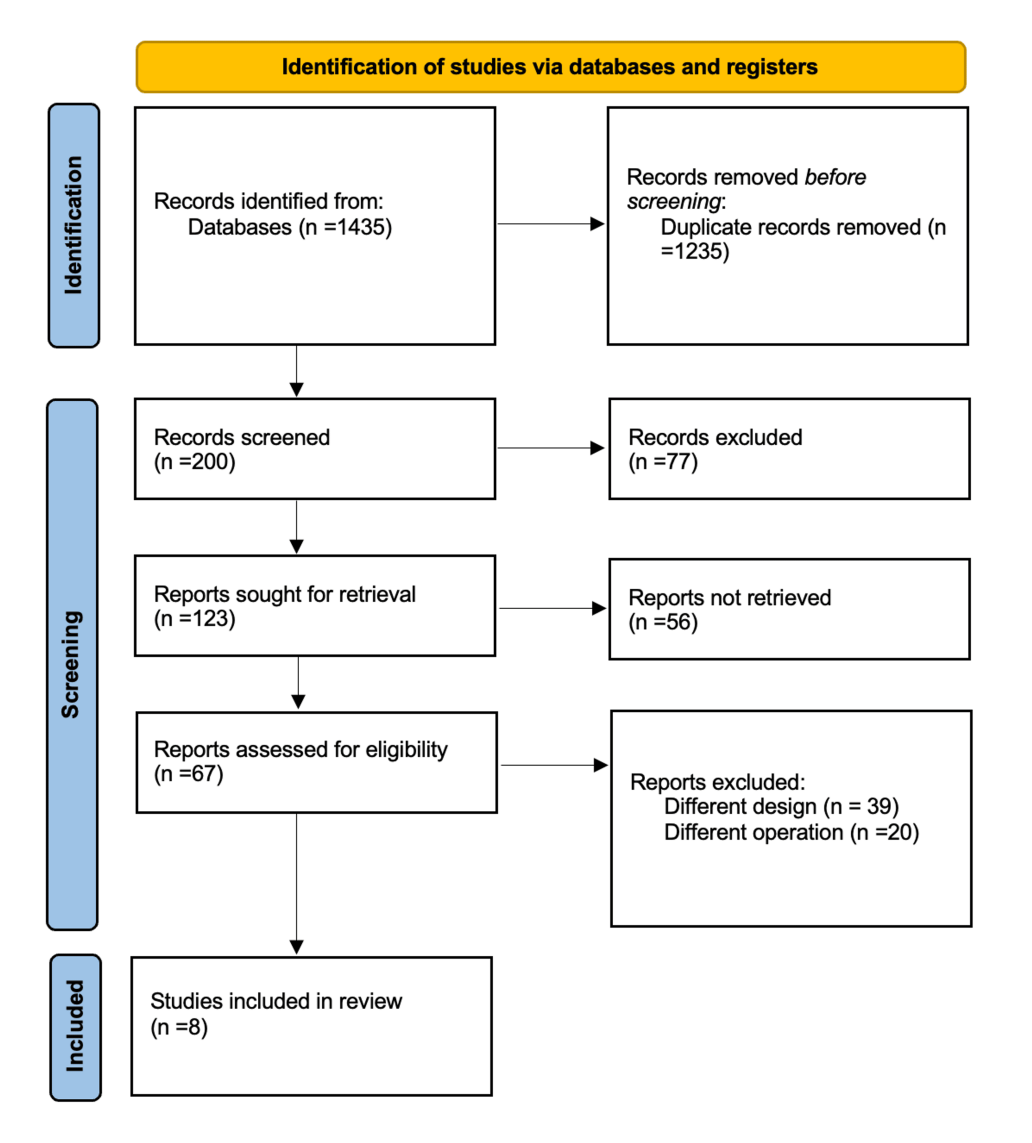
Preferred Reporting Items for Systematic Reviews and Meta-Analyses for study selection.

A total of 1,435 records were identified through database searches. After eliminating 1,235 duplicates, 200 unique studies remained for screening. Of these, 77 were excluded following title and abstract review. Full-text retrieval was attempted for 123 reports; however, 56 could not be accessed. The remaining 67 full-text articles were assessed for eligibility. After review, 58 were excluded (39 for having incompatible designs and 20 due to unrelated surgical procedures). Consequently, 8 RCTs were included in the final analysis. The complete study selection process is illustrated in Figure 1.

Summary of Included Studies

This review included eight RCTs published between 2011 and 2022, conducted in a range of countries such as the United Kingdom, India, China, Spain, Canada, Egypt, and Australia. The studies varied in size, with participant numbers ranging from 41 to 120. All of them compared the TAP block with TEA. Most trials provided details on patient age and demographics, with a fairly balanced mix of male and female participants overall, though a few studies didn’t report sex-specific data. The baseline summary of the included studies is presented in Table 1

**Table 1:** Baseline summary of the selected studies. TAP, transversus abdominis plane; TEA, thoracic epidural analgesia; RCT, randomized controlled trial; SD, standard deviation

Study ID	Study design	Area	Time	Population	Intervention	Population by intervention	Male	Female	Age, mean ± SD (years)
Niraj et al. (2014) [8]	RCT	United Kingdom	2014	70	TAP	35	19	16	66.7 ± 12.6
					TEA	35	22	13	64.4 ± 16.2
Xu et al. (2020) [9]	RCT	China	2020	120	TAP	60	35	25	60.4 ± 9.3
					TEA	60	36	24	61.4 ± 9.3
Tejedor et al. (2022) [10]	RCT	Spain	2022	43	TAP	23	Not found	Not found	65.6 ± 4.8
					TEA	20	Not found	Not found	65.9 ± 5.4
Ganapathy et al. (2015) [11]	RCT	Canada	2015	50	TAP	26	20	39	61.7 ± 10.8
					TEA	24	8	16	58.0 ± 12.2
Wahba et al. (2013) [12]	RCT	Egypt	2013	44	TAP	22	11	11	66.40 ± 48
					TEA	22	10	12	66.3 ± 5.4
Niraj et al. (2011) [13]	RCT	United Kingdom	2011	58	TAP	27	18	9	64 ± 11
					TEA	31	20	11	64 ± 11
Kadam et al. (2013) [14]	RCT	Australia	2013	41	TAP	22	14	8	63.8 ± 10.1
					TEA	19	14	5	60.9 ± 13.2
Wu et al. (2013) [15]	RCT	China	2013	56	TAP	27	18	9	60 ± 7
					TEA	29	20	9	61 ± 7

Risk-of-Bias Assessment

The overall risk of bias across the included studies was generally low. Most studies demonstrated a low risk in all assessed domains, particularly in the randomization process (D1), outcome measurement (D4), and reporting bias (D5).

However, a few studies raised some concerns in the timing of participant recruitment (D1b) and deviations from intended interventions (D2). These issues did not significantly compromise the integrity of the overall evidence, but they highlight the need for cautious interpretation of results from those specific trials.

**Figure 2:**
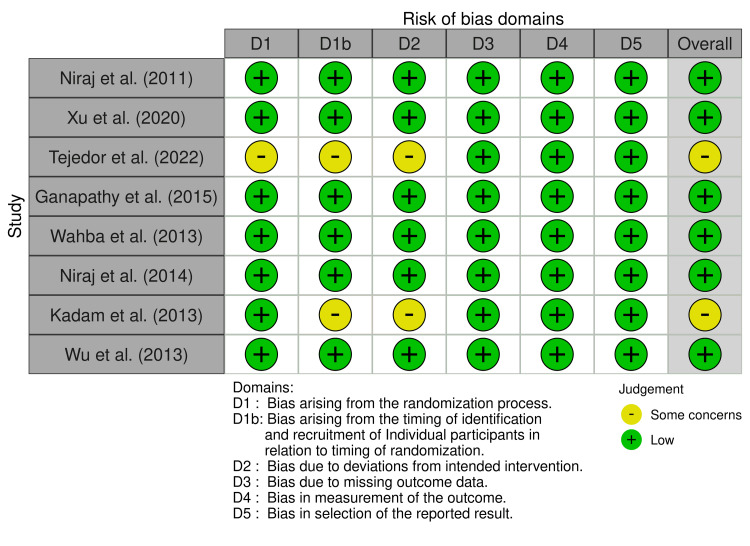
Risk-of-bias assessment across included randomized controlled trials. Studies included: [8,9,10,11,12,13,14,15].

In total, 6 out of 8 studies were judged to have low overall risk of bias, indicating a strong level of methodological quality among the included evidence. The risk-of-bias assessment is presented in Figure 2.

Primary Outcomes

**Figure 3:**
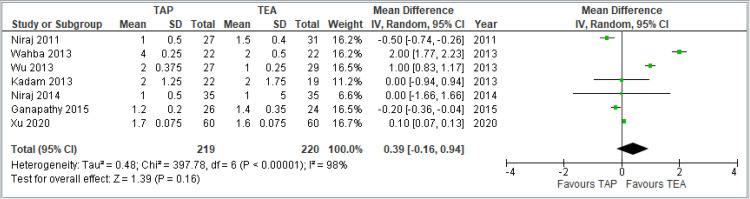
Forest plot comparing VAS pain scores at rest 24 hours postoperatively between TAP block and TEA. Studies included: [8,9,11,12,13,14,15]. TAP, transversus abdominis plane; TEA, thoracic epidural analgesia; VAS, visual analog scale; CI, confidence interval; SD, standard deviation

Pain scores at rest at 24 hours: Visual analog scale (VAS) was used for scoring the severity of pain. This forest plot summarizes seven studies comparing TAP block and TEA for pain at rest 24 hours postoperatively. The pooled MD was 0.26 (95% confidence interval (CI): -0.52 to 1.03), indicating no significant difference between TAP and TEA (*p* = 0.51). The CI crosses zero, suggesting statistical insignificance. Heterogeneity is high (*I*² = 99%), reflecting substantial variability across studies. Overall, no clear advantage was found for either technique in reducing pain at rest 24 hours after surgery. The combined results are shown in Figure 3.

**Figure 4:**
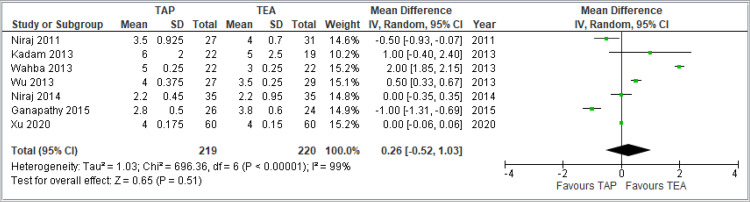
Forest plot comparing VAS pain scores during coughing at 24 hours postoperatively between TAP block and TEA. Included studies: [8,9,11,12,13,14,15] TAP, transversus abdominis plane; TEA, thoracic epidural analgesia; VAS, visual analog scale; CI, confidence interval; SD, standard deviation

Pain scores during coughing at 24 hours: This forest plot includes seven studies comparing TAP block and TEA for pain during coughing 24 hours after surgery. The pooled MD is 0.39 (95% CI: -0.16 to 0.94), indicating no statistically significant difference between the two techniques (*P* = 0.16). The CI crosses zero, suggesting a lack of definitive evidence favoring either approach. There is considerable heterogeneity (*I*² = 98%), pointing to variability among study designs, populations, or interventions. Despite some individual studies showing benefit, the overall analysis does not support a consistent advantage of one technique over the other for cough-related pain at 24 hours. The combined results are shown in Figure 4.

**Figure 5:**
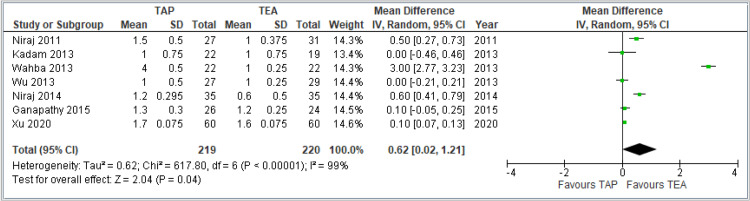
Forest plot comparing VAS pain scores at rest 48 hours postoperatively between TAP block and TEA. Included studies: [8,9,11,12,13,14,15]. TAP, transversus abdominis plane; TEA, thoracic epidural analgesia; VAS, visual analog scale; CI, confidence interval; SD, standard deviation

Pain scores at rest at 48 hours: This forest plot includes six studies comparing TAP block and TEA for pain at rest 48 hours after surgery. The pooled MD is 0.22 (95% CI: -0.40 to 0.85), indicating no significant difference between TAP and TEA (*P* = 0.48). The CI crosses zero, meaning the results are not statistically significant. Heterogeneity is high (*I*² = 99%), reflecting substantial variation among studies. This variability suggests caution in interpreting the pooled estimate, as clinical or methodological differences may influence the outcomes. In summary, there is no clear evidence favoring either TAP or TEA for pain control at rest 48 hours after surgery. The combined results are shown in Figure 5.

**Figure 6:**
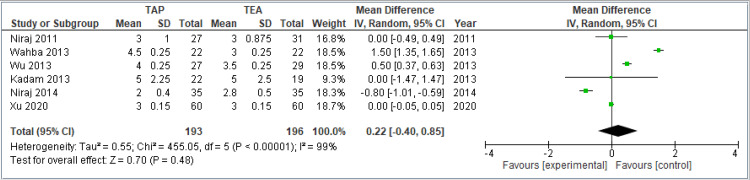
Forest plot comparing VAS pain scores during coughing at 48 hours postoperatively between TAP block and TEA. Included studies: [8,9,12,13,14,15]. TAP, transversus abdominis plane; TEA, thoracic epidural analgesia; VAS, visual analog scale; CI, confidence interval; SD, standard deviation

Pain scores during coughing at 48 hours: This forest plot includes seven studies comparing TAP block and TEA for cough-related pain at 48 hours post-surgery. The overall pooled MD is 0.62 (95% CI: 0.02-1.21), favoring TEA, with statistical significance (*P* = 0.04). The CI does not cross zero, indicating a small but meaningful advantage of TEA over TAP for this outcome. Despite the positive finding, heterogeneity remains high (*I*² = 99%), highlighting variability across studies, which could be due to differences in protocols, surgical types, or patient populations. In summary, TEA appears to offer better pain relief during coughing at 48 hours postoperatively compared to TAP, though the significant heterogeneity warrants cautious interpretation. The combined results are shown in Figure 6.

Secondary Outcomes

**Figure 7:**
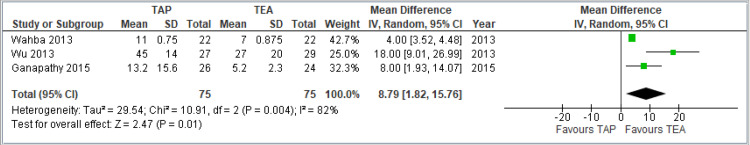
Forest plot comparing total postoperative opioid (morphine equivalent) consumption between TAP block and TEA. Included studies: [11,12,15]. TAP, transversus abdominis plane; TEA, thoracic epidural analgesia; VAS, visual analog scale; CI, confidence interval; SD, standard deviation

Postoperative opioid (morphine) consumption (in milligrams): This forest plot compares postoperative opioid consumption between TAP block and TEA based on data from three studies. All studies consistently show higher opioid use in the TAP group, with significant MDs favoring TEA. The pooled MD is 8.79 (95% CI: 1.82-15.76), indicating significantly lower opioid consumption in the TEA group (*P* = 0.01). Since the CI does not cross zero, the difference is statistically significant. Moderate-to-high heterogeneity is present (*I*² = 82%), suggesting some variability across studies. This could be due to differences in opioid measurement methods, surgical procedures, or dosing protocols. In summary, TEA appears more effective than TAP block in reducing postoperative opioid requirements, though the observed heterogeneity should be considered when interpreting these results. The combined results are shown in Figure 7.

**Figure 8:**
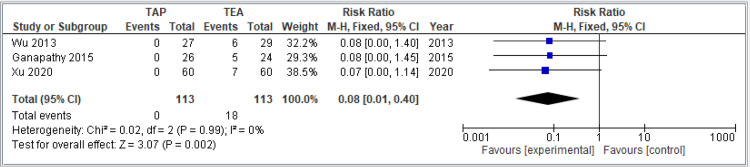
Forest plot comparing the incidence of postoperative hypotension between TAP block and TEA. Included studies: [9,11,15]. TAP, transversus abdominis plane; TEA, thoracic epidural analgesia; VAS, visual analog scale; CI, confidence interval; SD, standard deviation

Postoperative hypotension: This forest plot analyzes the incidence of postoperative hypotension in three studies comparing TAP block and TEA. None of the patients in the TAP groups experienced hypotension, while several cases occurred in the TEA groups. The pooled RR is 0.08 (95% CI: 0.01-0.40), indicating a significantly lower risk of hypotension with TAP block compared to TEA (*P* = 0.002). This effect is statistically significant, with the CI well below 1. There is no statistical heterogeneity across the studies (*I*² = 0%), suggesting consistent findings despite differences in study design or populations. In summary, the TAP block is associated with a significantly lower risk of postoperative hypotension compared to TEA, with consistent results across studies. The combined results are shown in Figure 8.

**Figure 9:**
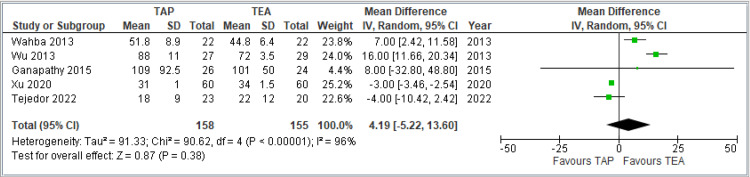
Forest plot comparing the average time to pass first flatus (in hours) between TAP block and TEA. Included studies: [9,10,11,12,15]. TAP, transversus abdominis plane; TEA, thoracic epidural analgesia; VAS, visual analog scale; CI, confidence interval; SD, standard deviation

Average time to pass first flatus (in hours): This forest plot includes five studies comparing the effect of TAP block versus TEA on time to pass first flatus, a marker of postoperative bowel recovery. The pooled MD is 4.19 hours (95% CI: -5.22 to 13.60), indicating no statistically significant difference between the two techniques (*P* = 0.39). The wide CI crossing zero confirms this. There is substantial heterogeneity (*I*² = 96%), suggesting major variation across studies, potentially due to differences in surgical procedures, patient populations, or postoperative care protocols. In summary, there is no conclusive evidence that either TAP block or TEA provides superior outcomes in terms of time to first flatus. The marked heterogeneity underscores the need for cautious interpretation. The combined results are shown in Figure 9.

**Figure 10:**
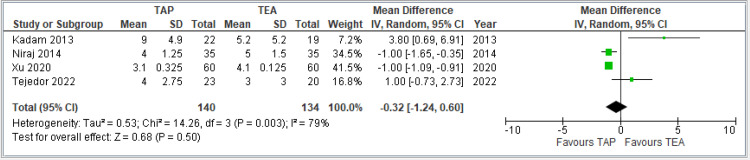
Forest plot comparing length of hospital stay (in days) between TAP block and TEA. Included studies: [8,9,10,14]. TAP, transversus abdominis plane; TEA, thoracic epidural analgesia; VAS, visual analog scale; CI, confidence interval; SD, standard deviation

Length of hospital stay (in days): This forest plot includes four studies comparing TAP block and TEA in terms of hospital stay duration. The pooled MD is -0.32 days (95% CI: -1.24 to 0.60), indicating no statistically significant difference between TAP and TEA (*P* = 0.50). The CI crosses zero, suggesting uncertainty about any true effect. Moderate heterogeneity is present (*I*² = 79%), indicating some variability among study results, possibly due to differences in surgical practices, discharge criteria, or patient populations. In summary, the current evidence does not support a significant difference in hospital stay duration between TAP block and TEA. The combined results are shown in Figure 10.

Discussion

This meta-analysis evaluated the comparative effectiveness of TAP block and TEA in adult patients undergoing abdominal surgery. The results indicate that both techniques provide comparable pain control at rest and during coughing within the first 48 hours postoperatively. These findings are particularly valuable given the growing interest in regional anesthesia techniques that aim to balance efficacy with safety and ease of administration [16].

Although no statistically significant differences were observed in pain scores at 24 or 48 hours at rest, or 24 hours during coughing, TEA showed a slight advantage in reducing pain during coughing at 48 hours. This difference may be attributed to TEA’s ability to provide both somatic and visceral analgesia, while TAP block primarily affects somatic nerves of the anterior abdominal wall. Pain during coughing can often reflect deeper, visceral discomfort, which TEA, due to its neuraxial mechanism of action, is better equipped to address [17]. However, the differences were modest, and TAP block still achieved meaningful analgesia in most studies, indicating that it is a valid alternative, particularly in settings where the use of epidural analgesia is limited [18].

A more notable finding in this analysis is the significant reduction in opioid consumption among patients receiving TEA. This is consistent with the broader analgesic coverage of TEA, which allows for continuous delivery and enhanced opioid-sparing effects. However, this benefit is not without trade-offs, as TEA was associated with a significantly higher incidence of postoperative hypotension, a consequence of its blockade of sympathetic nerve fibers. In contrast, TAP block demonstrated virtually no episodes of hypotension, highlighting its peripheral mechanism, which limits cardiovascular effects [19]. This safety profile makes TAP block particularly attractive for patients with cardiovascular comorbidities or for surgeries where hypotension might pose a higher risk [20].

Despite the belief that TEA enhances bowel recovery through sympathetic inhibition, our findings did not reveal a significant difference between TAP and TEA in the time to pass first flatus. Similarly, hospital stay duration did not differ significantly between the two groups. This could be attributed to the influence of modern Enhanced Recovery After Surgery (ERAS) protocols and multimodal analgesia strategies that aim to standardize postoperative recovery across analgesic techniques. The absence of a significant difference in these outcomes may suggest that ERAS protocols, which include early mobilization and a combination of different pain management methods, reduce the gap between these two analgesic techniques in terms of recovery [21]. The overall speed of recovery may thus depend more on the implementation of these protocols than on the choice of analgesic technique.

While these results offer valuable insights, some limitations must be acknowledged. There was considerable heterogeneity in some outcomes, particularly in pain scores and opioid consumption, likely due to variations in TAP block techniques (e.g., subcostal versus posterior approaches), differences in local anesthetic agents and dosages, as well as variation in surgical types and postoperative care protocols. Some included studies had small sample sizes, and a few reported unclear or incomplete methodology, although the overall risk of bias was low across most studies [22]. This heterogeneity suggests that the choice of technique, type of surgery, and institutional protocols can influence the outcomes, and future research should aim to standardize methods and improve reporting.

From a clinical standpoint, the findings from this meta-analysis strongly support the use of TAP block as a safe and effective alternative to epidural analgesia for abdominal surgeries. Although TEA may offer superior visceral pain control and opioid-sparing benefits, TAP block presents itself as a simpler, safer option, particularly in terms of its cardiovascular safety profile. These advantages make TAP block an attractive choice, especially in patients with contraindications to neuraxial anesthesia or in resource-limited settings where epidural analgesia might be less feasible [23]. Furthermore, the lower incidence of hypotension with TAP block provides an additional safety benefit in these settings, where managing blood pressure is crucial [16].

Future research should focus on standardizing TAP block techniques and exploring their effects in specific surgical populations, particularly those with higher risks or unique clinical needs. Additionally, studies examining long-term outcomes, including functional recovery, quality of life, and patient satisfaction, would further inform clinical decision-making. Long-term data on pain management, recovery, and postoperative complications are essential to fully evaluate the comparative benefits of TAP block versus TEA [24]. Until more definitive evidence emerges, the choice between TAP block and TEA should be based on individual patient characteristics, the nature of the surgical procedure, and institutional capabilities and expertise [25].

## Conclusions

This meta-analysis demonstrates that TAP block provides postoperative analgesia comparable to TEA for abdominal surgeries, particularly in terms of pain control at rest and during coughing within the first 48 hours. While TEA offers advantages in reducing opioid consumption and improving cough-related pain at 48 hours, it is associated with a significantly higher risk of hypotension. TAP block, by contrast, offers a favorable safety profile, making it a valuable alternative in patients where epidural use may be contraindicated or less desirable. Given the comparable efficacy and improved hemodynamic stability, TAP block can be considered a reliable component of multimodal analgesia, especially in enhanced recovery pathways. Further high-quality trials are needed to confirm these findings and explore long-term outcomes and cost-effectiveness.
